# TEM sample preparation of lithographically patterned permalloy nanostructures on silicon nitride membranes

**DOI:** 10.3762/bjnano.15.1

**Published:** 2024-01-02

**Authors:** Joshua Williams, Michael I Faley, Joseph Vimal Vas, Peng-Han Lu, Rafal E Dunin-Borkowski

**Affiliations:** 1 Ernst Ruska-Centre for Microscopy and Spectroscopy with Electrons, Forschungszentrum Jülich, 52425 Jülich, Germany; 2 Faculty of Engineering, University Duisburg-Essen, 47057 Duisburg, Germany

**Keywords:** electron holography, Lorentz transmission electron microscopy, magnetic imaging, nanodisk, nanofabrication, permalloy

## Abstract

We have prepared ferromagnetic nanostructures intended for the investigation of high-frequency magnetization dynamics in permalloy (Py) nanodisks using Lorentz transmission electron microscopy (LTEM) and electron holography. Py nanodisks were fabricated on thin silicon nitride (SiN) membranes using three different fabrication methods: lift-off, ion beam etching (IBE), and stencil lithography. They were further analyzed using different instruments, including scanning electron microscopy, LTEM, and electron holography. A bilayer of positive PMMA resist was utilized in the first fabrication method to form an undercut structure that guarantees a clean lift-off procedure. The second approach used dry etching with an Ar beam to etch a thin Py film, while an electron-beam-patterned negative resist mask kept the desired structure. In the third process, nanostencils (shadow masks) with submicrometer apertures were milled on SiN membranes using a focused ion beam. Furthermore, we have developed a new TEM sample preparation method, where we fabricated Py nanostructures on a bulk substrate with a SiN buffer layer and etched the substrate to create a thin SiN membrane under the Py nanostructure. Finally, we observed the vortex dynamics of the Py nanodisk under magnetic fields using LTEM and off-axis electron holography. A correlation between preparation methods and the properties of the Py nanostructures was made.

## Introduction

The ability to study the spatial distribution of magnetization in ferromagnetic nanostructures is important for developing nanoelectronics, particularly for data storage and information processing. A vortex spin configuration has been observed in Py nanodisks [1–2] with independent polarity and helicity [3]. Since then, many studies have been done on manipulating magnetic vortices inside Py nanodisks using micromagnetic simulations [4–6] and a variety of magnetic measurement techniques including magnetic force microscopy [7], transmission electron microscopy (TEM) [1,8–11], scanning transmission X-ray microscopy [12–13], and magneto-optical Kerr effect microscopy [14–15]. Possible applications of Py nanodisks were proposed for zero-hysteresis magnet sensors, magnetic logic devices, and data storage [16]. Py is a nickel–iron alloy (80 atom % Ni and 20 atom % Fe) that has a small coercive field (*H*_c_) [17] and low magnetostriction (λ_s_) [18], as well as high permeability and high saturation magnetization (*M*_s_) [19].

TEM offers high spatial resolution for magnetic imaging. TEM-based magnetic imaging techniques such as Lorentz microscopy and electron holography, along with simultaneous structural and chemical characterization techniques such as electron diffraction, 4D STEM, and energy-dispersive X-ray (EDX) and electron energy loss spectroscopy (EELS), enable a correlative characterization to investigate magnetic information down to the nanometer/atomic scale. However, the corresponding samples need to be prepared on electron-beam-transparent membranes, which are very fragile and can easily break during standard lithography procedures. Although a lift-off approach has been demonstrated [20], alternative methods may be advantageous in terms of structural resolution, process simplicity, and the absence of resist residues [21]. We have fabricated ferromagnetic nanodisks on a conventional TEM grid from TedPella^®^ using three different fabrication methods.

In the first method, a bilayer of positive PMMA resist yielded an undercut structure. The resist was patterned using an electron beam, which offers higher resolution than other sources (e.g., UV light) because of the smaller wavelength of electrons. Since the use of an ultrasonic bath will destroy the free-standing membrane, the undercut must be deliberately made larger to ensure a clean lift-off process. The larger undercut is realized by multi-dose exposure, which consists of two parts: The main exposure is for patterning the nominal structure, and an additional exposure is for patterning the outline of the nominal structure. This additional exposure is performed with a lower dose than the main exposure so that it does not induce chain scission in the top resist layer, but only in the bottom resist layer, which is more sensitive. The result of the multi-dose exposure was controlled by observing a cross section of the developed bilayer resist using a SEM in snapshot mode to avoid melting of the PMMA resist. The second approach involved etching a thin Py film with an ion beam while preserving the intended structure with an electron-beam-patterned negative resist mask. Redeposition of etched material was found to construct fences at the edges of the structures. Fences and edge roughness from the imperfect lift-off process were reported to influence the magnetic properties of nanostructures [22]. The third method, stencil lithography, makes use of a shadow mask, which was fabricated by milling submicrometer apertures on a conventional TEM grid using a focused ion beam. This method avoids the resist-based fabrication, which is common in preparing nanodisk samples for TEM [8,20].

We have also developed a method of sample preparation for patterned nanostructures starting from a bulk substrate. This method is versatile and might be useful for more complicated lithographically patterned nanostructures to be examined using TEM. The results of the fabrication methods mentioned above were examined using SEM. This is important because the structural information (disk dimensions and deformation from fences) later correlates to the magnetic properties. A magnetic vortex configuration occurs only under the right diameter/thickness ratio, otherwise either a single or multiple magnetic domains will appear.

After Py nanodots of various sizes were fabricated, we used Lorentz transmission electron microscopy (LTEM) and off-axis electron holography to study their magnetic domain structure. The microscope is operated in a magnetic-field-free mode. In this case, the objective lens is turned off, and the Lorentz lens is used instead to focus the electron beam onto the back focal plane. As the electron beam passes through the sample, the in-plane sample magnetization exerts a Lorentz force onto the electron beam, which deflects the beam. The force on each electron in the beam is given by


[1] F=−e(v×B),

where *F* is the force, *e* is the charge, *v* is the relativistic velocity of the electron beam, and *B* is the magnetic field exerted by the sample.

In the case of a vortex structure, the electron beam is deflected by the circularly oriented magnetic fields. The magnetic contrast can hardly be observed when the image is in-focus but becomes more visible when the image is defocused. On one side of the focus, the magnetization of the vortex deflects the electron beams inwards, which then overlaps and results in a white contrast in the center. On the other side of the focus, the beam will be deflected outwards leaving an empty area and, therefore, a dark contrast in the center [23].

Off-axis electron holography is obtained from the interference (holograms) of the electron wave modulated by the magnetic sample and a coherently tilted reference plane wave. The intensity of the hologram can be represented in the form of


[2] $$\begin{array}{c} I_{\text{hol}}\left(r\right)={\left|\Psi_{\text{i}}\left(r\right)+\exp\left[2\pi \text{i }q\cdot r\right]\right|}^{2}\\ =1+A_{\text{i}}{}^{2}\left(r\right)+2A_{\text{i}}\left(r\right)\text{cos}\left[2\pi \text{i }q\cdot r+\varphi_{\text{i}}\left(r\right)\right], \end{array}$$

where Ψ_i_(**r**) stands for the electron wavefunction in the image plane i with amplitude *A*_i_ and phase φ_i_, **r** is a two-dimensional vector in the sample plane, and **q** is the two-dimensional reciprocal space vector related to the tilt of the reference wave. Note that the phase φ_i_(**r**) is now separated in the third term inside the cosine; it can be retrieved by taking the fast Fourier transform (FFT) of the intensity [24]. The phase shift can then be used to recover the in-plane magnetic information inside the sample.

## Results and Discussion

### Fabrication on a commercial SiN membrane

#### Lift-off

**Figure 1:**
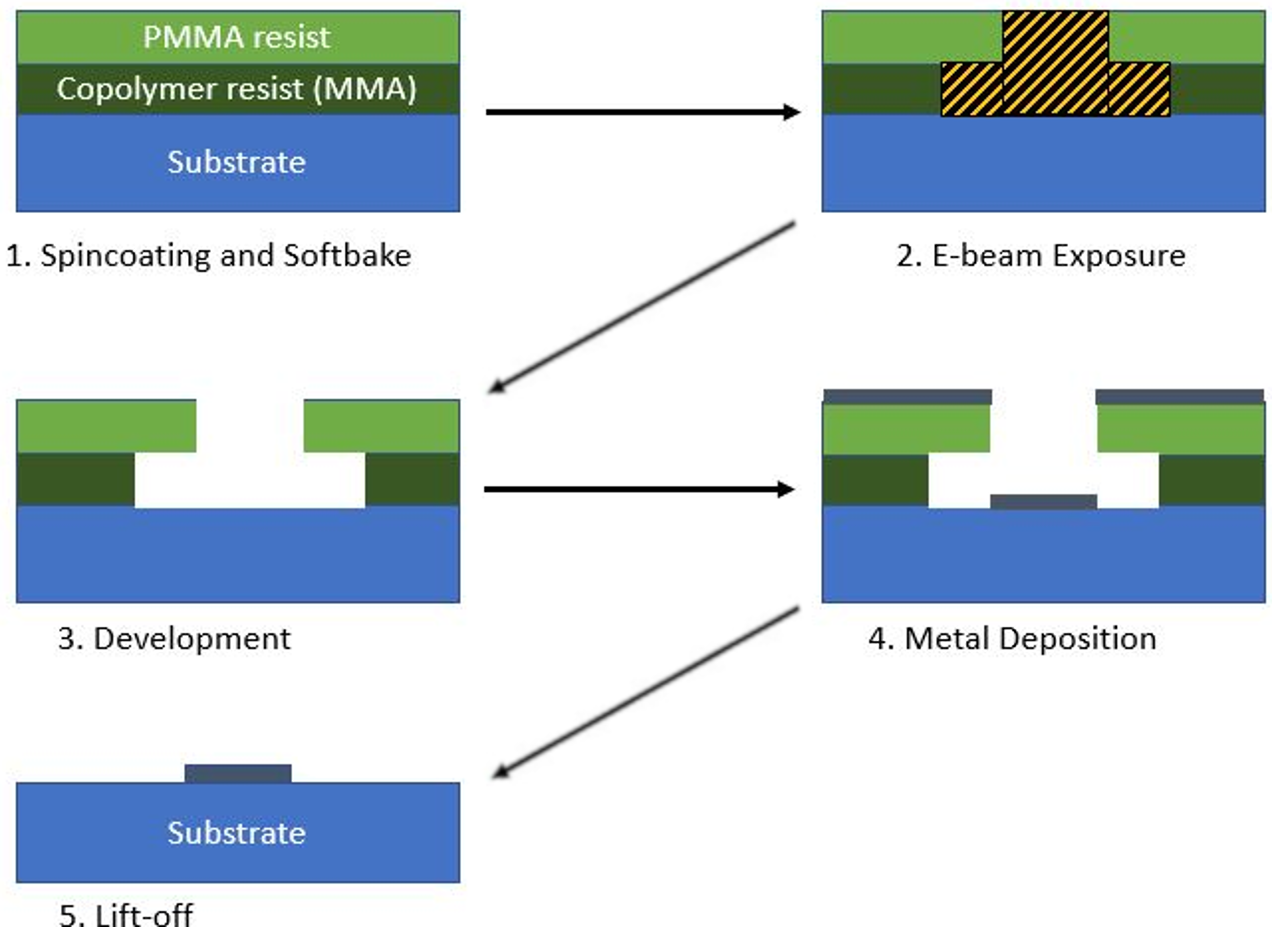
Lift-off fabrication process.

**Figure 2:**
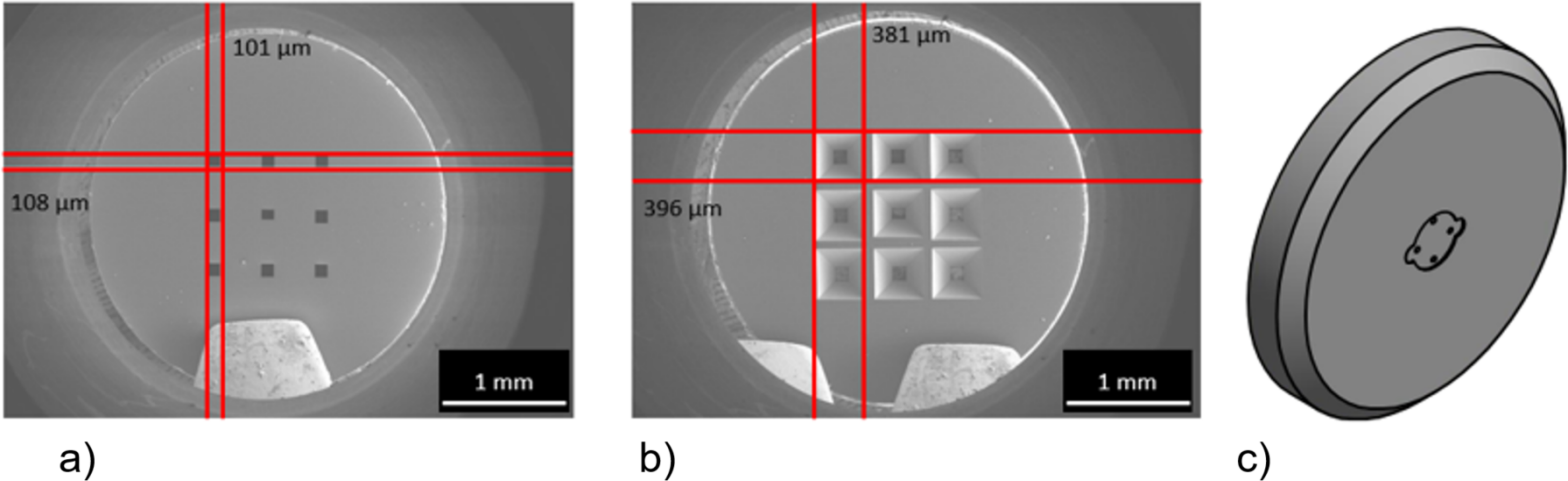
SEM images of the 3 mm TEM window grid that provides nine windows of free-standing 50 nm thick SiN membrane. (a) Front side and (b) back side. (c) Spin coating adapter for the 3 mm TEM grid.

The lift-off procedure is described in Figure 1. We use PMMA and its copolymer as a positive resist to create a bilayer resist. The copolymer (AR-P 617.08) is a methyl methacrylate and methacrylic acid copolymer dissolved in 1-methoxy-2-propanol with a solid content of 8%. Its viscosity is 36 mPa·s. After spinning at 4000 rpm and baking at 200 °C for 25 min, it has a thickness of around 500 nm. To spin coat a 3 mm TEM grid (Figure 2a,b), we used a special adapter (Figure 2c). The high baking temperature and the relatively long baking duration were chosen because the copolymer must be solid to prevent dissolution by the PMMA layer. In addition, the baking temperature can control the sensitivity of the copolymer. The higher the baking temperature of the copolymer, the more sensitive the copolymer gets. This is because more anhydride 6-rings form, which break apart more easily than the aliphatic chain remainder during electron beam exposure [25]. AR-P 679.04 is polymethylmethacrylate (PMMA) dissolved in ethyl lactate with a solid content of 4%. Its molecular weight is 950,000, and it has a high resolution as well as a low sensitivity. Its viscosity is 16.4 mPa·s. After spinning at 4000 rpm and baking at 180 °C for 5 min, it has a thickness of around 300 nm. The exposure is carried out using an electron beam lithography system Vistec EBPG 5000+ operating at 100 kV. Working on a thin transparent membrane also allows for high-resolution patterning since there is less electron scattering during exposure [26].

The purpose of applying two layers of resist is to create a large undercut by using a bottom layer that is more sensitive than the top layer. This prevents the unwanted deposition of metal that sticks to the side of the resist after lift-off. A larger undercut was achieved on multilayer resist with the help of multiple exposures [27]. The idea is to expose the resist layer by layer: The bottom resist was deposited and exposed. Then, aluminium was deposited as a spacer between the bottom and top layer to prevent the top layer from dissolving the lower one. Next, the top resist layer was deposited and exposed to the nominal size. The structure was developed from top to bottom including removal of the Al spacer. The development of the bottom layer, in other words the size of the undercut, is controlled only by the development time. In a more recent study, the Al spacer was omitted, and the development was done with one solution since the bilayer resist is made from PMMA and its copolymer [28]. The process is quite time-consuming since the exposures are done layer by layer.

**Figure 3:**
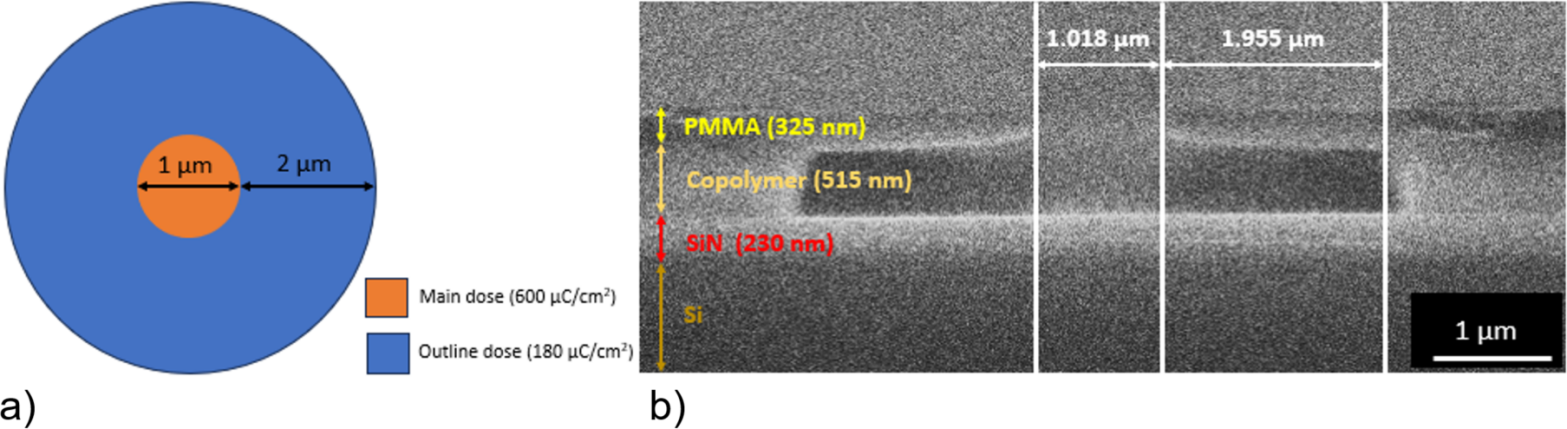
(a) Electron beam exposure scheme. (b) Cross section of developed resist with multi-dose exposure on a bulk substrate taken by SEM in snapshot mode.

Considering the two techniques, a one-time exposure is possible with the help of high accelerating voltage during electron beam exposure. In this process, rather than doing one resist deposition and exposure after another, the layer selectivity is controlled by the electron beam dose and the sensitivity of the two layers. Only in the copolymer (higher sensitivity) the chain scission reaction occurs at low doses; at higher doses, both layers were exposed. The exposure scheme is given in Figure 3.

The doses were chosen by considering that the copolymer is 2–3 times more sensitive than PMMA [25]. If the exposure dose is too small, then the undercut will not develop; if it is too high, then PMMA will dissolve in a larger area than the nominal diameter. It takes approximately 15 min development time to get 2 µm undercut that is shown in Figure 3b.

**Figure 4:**
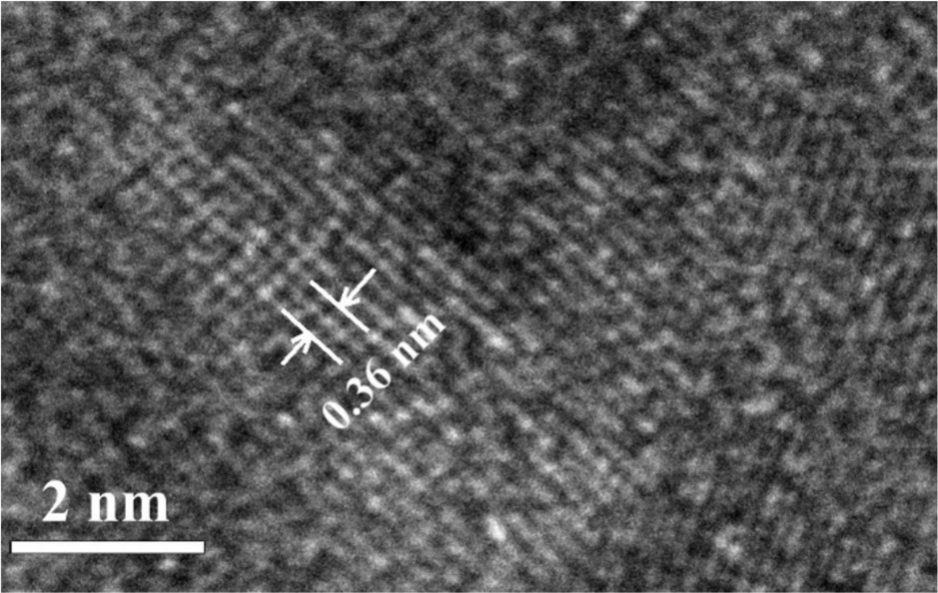
HRTEM image of a Py nanodisk. The grain sizes are around 6 nm.

Once the resist was developed, we deposited a 50 nm thick layer of Py using magnetron sputtering through the resist aperture. We used DC magnetron sputtering in a pure Ar environment at a pressure of 1 Pa to deposit Py at room temperature. The effective permalloy target had a diameter of 8 mm. The sputtered material almost forms a parallel beam when it approaches the substrate at a target–substrate distance of around 8 cm. The sputtered film was investigated under HRTEM. It was revealed that the film is polycrystalline with a lattice spacing of 0.36 nm (Figure 4), which correlates to the lattice constant of Py.

**Figure 5:**
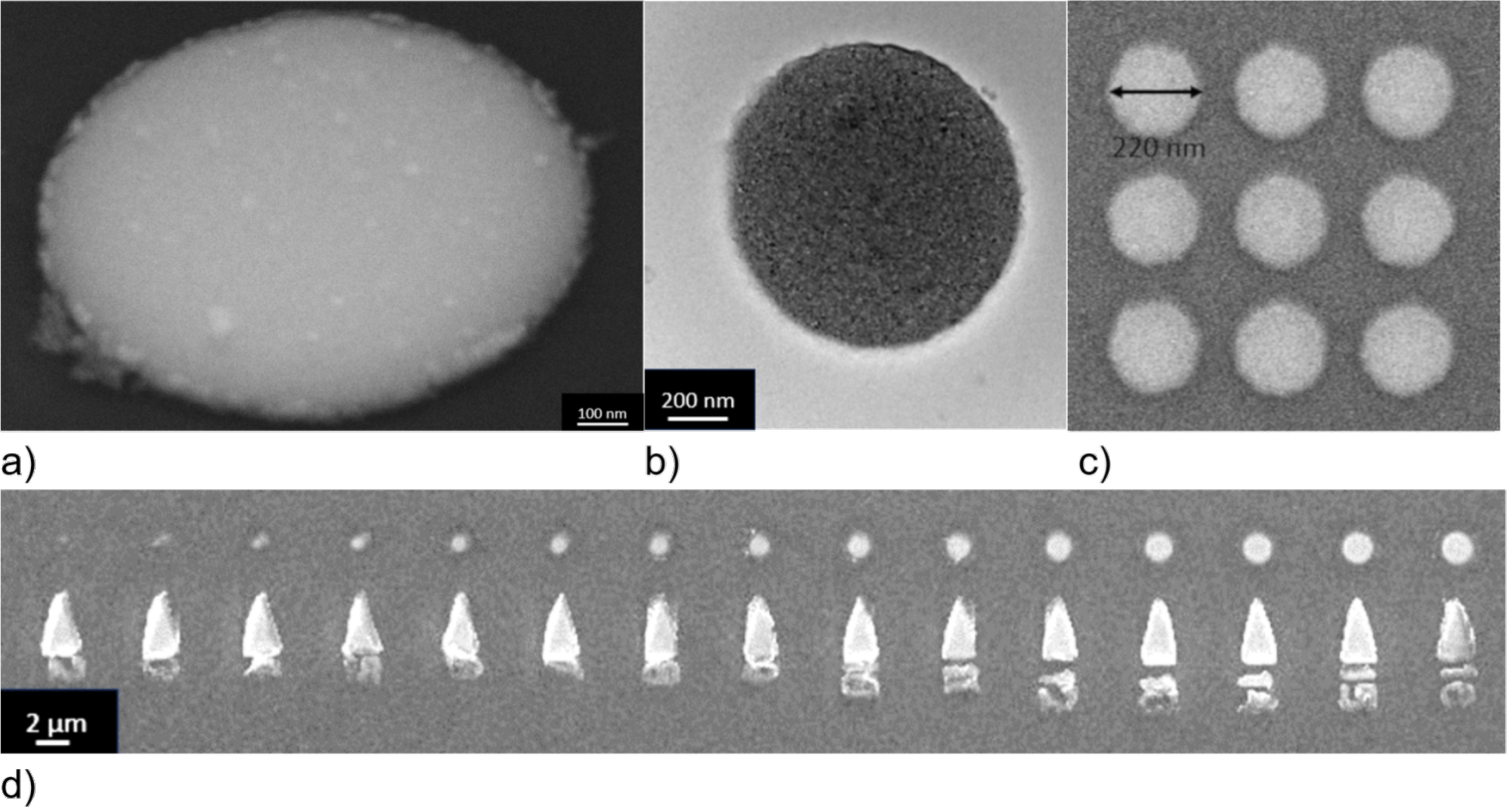
(a) SEM image of a Py disk (1 µm diameter, 50 nm thickness) at a tilt angle. (b) TEM image of a Py disk with 1 µm diameter. (c) SEM image of a 200 nm disk array with 50 nm spacing. (d) SEM image of Py nanodisks of different sizes.

The lift-off technique allows for the fabrication of arrays of 220 nm disks spaced 80 nm apart (see Figure 5c). With the help of the undercut, there is significantly less debris of metal after lift-off (Figure 5a). In comparison, the arrows on the lower part of Figure 5d demonstrate the lift-off result without a big undercut, which leads to fences of Py deposited on the sides of the resist. There are, however, a few limitations to consider: The spin-coated resist may be inhomogeneous (edge bead effect) on smaller substrates, reducing the region where high-quality structures may be obtained. Furthermore, one cannot deposit metals at high temperature, and one has to establish a good thermal contact during metal deposition to prevent the resist mask from melting as the substrate temperature is above the glass transition temperature of the resist.

#### Ion beam etching

**Figure 6:**
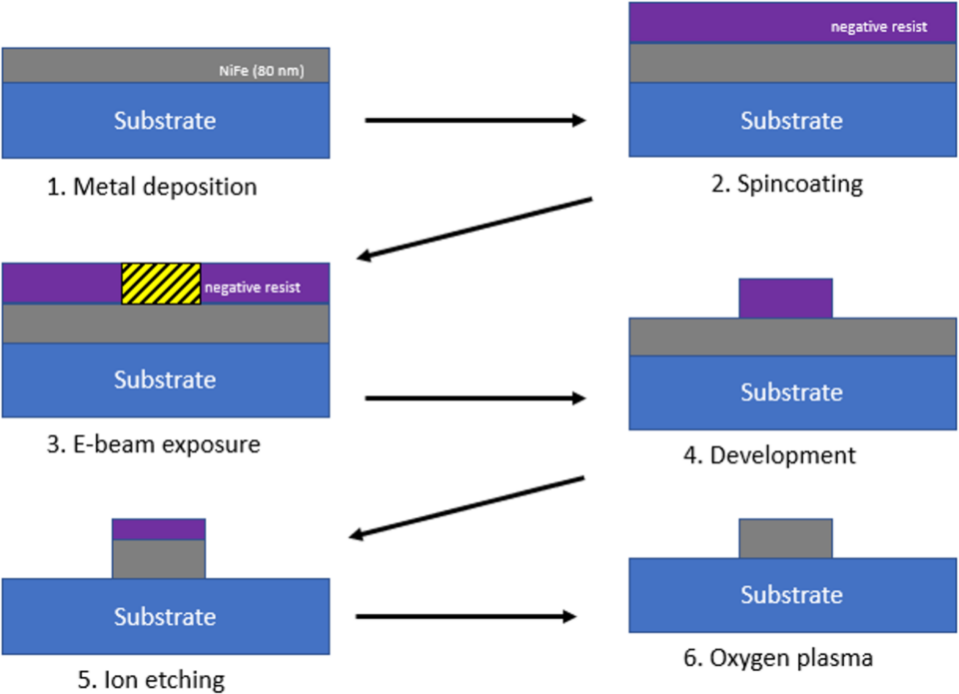
Ion beam etching process.

The IBE process (Figure 6) is as follows: The first step is to deposit Py on the substrate; then a negative resist is spin-coated on top. The resist used is AZ^®^ nLof 2020 diluted with AZ^®^ EBR solvent. The producers describe this as a photoresist (UV), but it is also compatible with electron beams. It is spun on top of Py at 4000 rpm and baked at 110 °C for 1 min. The resulting thickness is around 448 nm for 1:1 diluted resist and around 203 nm for 1:2 diluted resist. The resist is exposed to 120 µC/cm^2^ at 100 kV and then post-exposure baked at 110 °C for 2 min. The development is done by submerging the sample in AZ^®^ 726MIF containing 2.38% tetramethylammonium hydroxide (TMAH) for 20 s.

**Figure 7:**
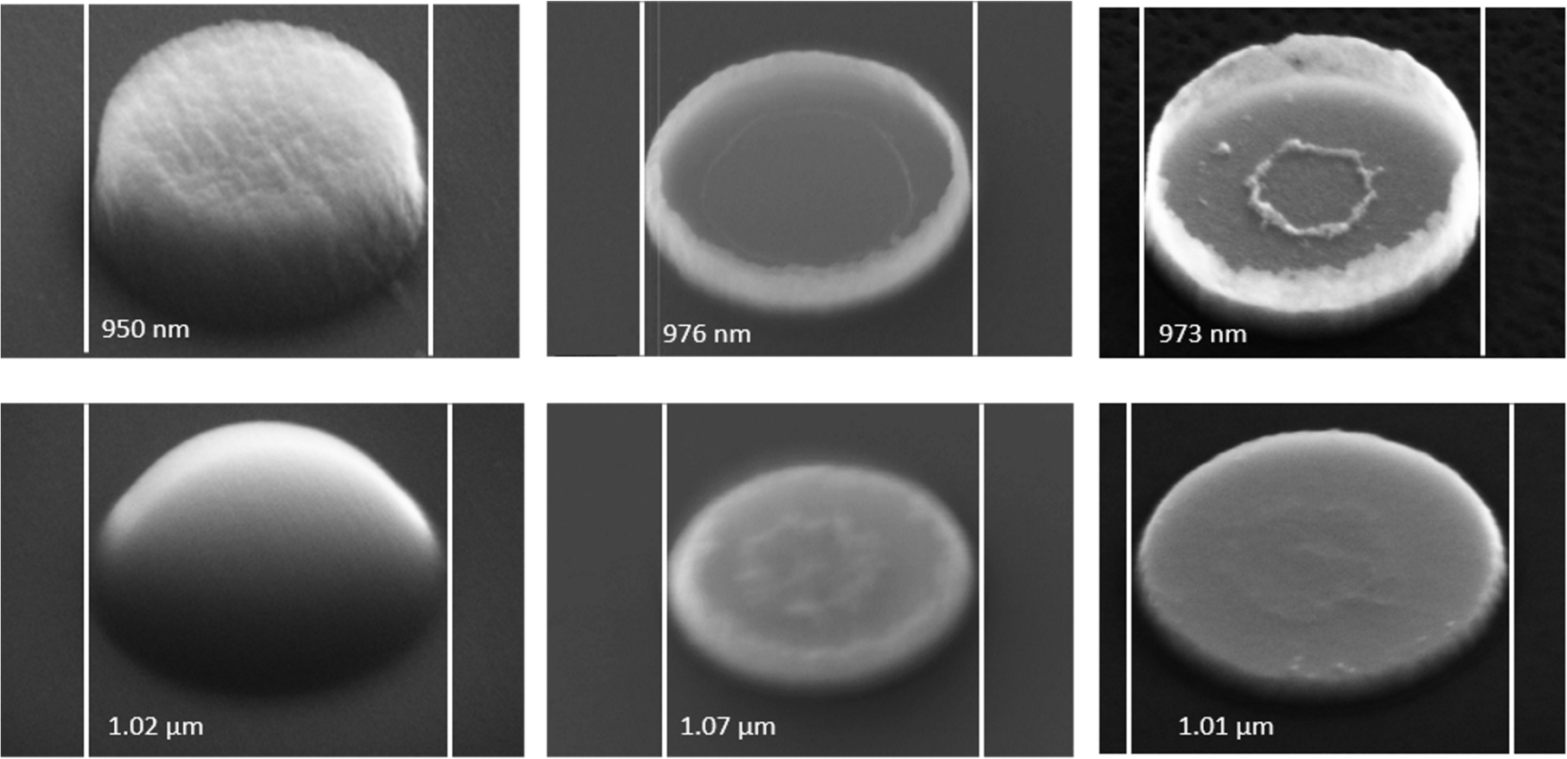
Comparison between normal patterned resist (top) and reflowed resist (bottom). From left to right: patterned resist, nanodisk after IBE, and nanodisk after plasma cleaning.

Once the resist is developed, IBE is performed. Since the ions diverge as they travel, the substrate is rotated to obtain a uniform ion flux on the substrate and a uniform etching rate between inner and outer sides. The highest etching rate can be achieved when the substrate is tilted at 45° since the etching rate of the primary beam is much bigger than the redeposition rate of etched materials. As observed in Figure 7, there was redeposition of etched material along the edge of the resist. This can be avoided by taking an additional step before etching the Py: The resist is heated at 120 °C for 5 min to reflow the resist and to create a meniscus shape, thus, decreasing the redeposition at the edge of the resist during etching.

**Figure 8:**
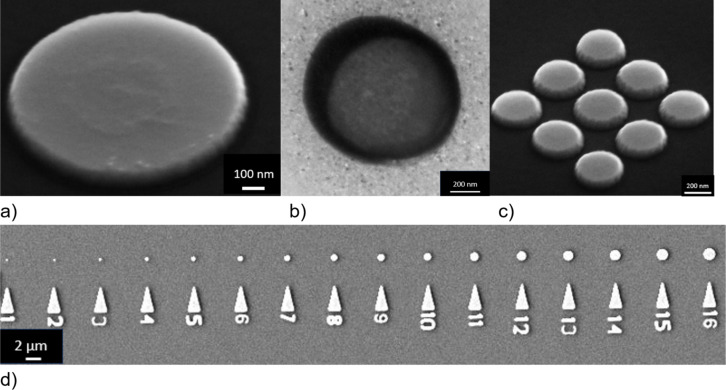
(a) SEM image of a Py disk (1 µm diameter, 80 nm thickness) at a tilt angle. (b) TEM image of a Py disk with 1 µm diameter. (c) SEM image of a 200 nm disk array with 50 nm spacing. (d) SEM images of nanodisk with different sizes.

The advantage of IBE are the well-defined structures with good edge sharpness (Figure 8). This technique offers high resolution for structures down to 200 nm, and dense structures with spacings as small as 50 nm can be created. Another advantage is the ability to deposit metal at high temperatures as the resist mask is applied after the metal deposition. However, there are also some drawbacks to consider. The sample is physically etched by argon bombardment, which results in a non-selective etching of the material. Non-selective etching means that not only Py is etched but also the resist and the SiN membrane. Areas around the nanodisks are thicker and rougher because of the lower etch rate near the structures and the redeposition of Py. In addition, the much thinner areas of the membrane away from the nanostructures can affect the overall mechanical stability of the membrane. Dry etching is generally better suited for bulk substrate applications as discussed in section “Preparation of nanostructures starting from a bulk substrate”.

#### Stencil lithography

Stencil lithography was implemented back in 1978 to fabricate thin-film Josephson devices [29]. The principle behind stencil lithography is to deposit materials onto the substrate through the aperture of a hard mask. Rather than placing the mask directly on the substrate and then removing it, as in traditional resist-based fabrication, stencil lithography employs a separate mask that is later aligned on the substrate and retains its aperture after the pattern transfer. This technique has advantages including simplicity in process, reusable masks, and the absence of resist masks. The latter eliminates common challenges associated with resist such as the edge bead problem and resist melting during deposition. This approach is ideal for applications on small substrates where spin coating of a homogeneous resist layer is difficult. This technique is particularly suitable for TEM application because TEM grids have the SiN membrane that can be used as a hard mask.

**Figure 9:**
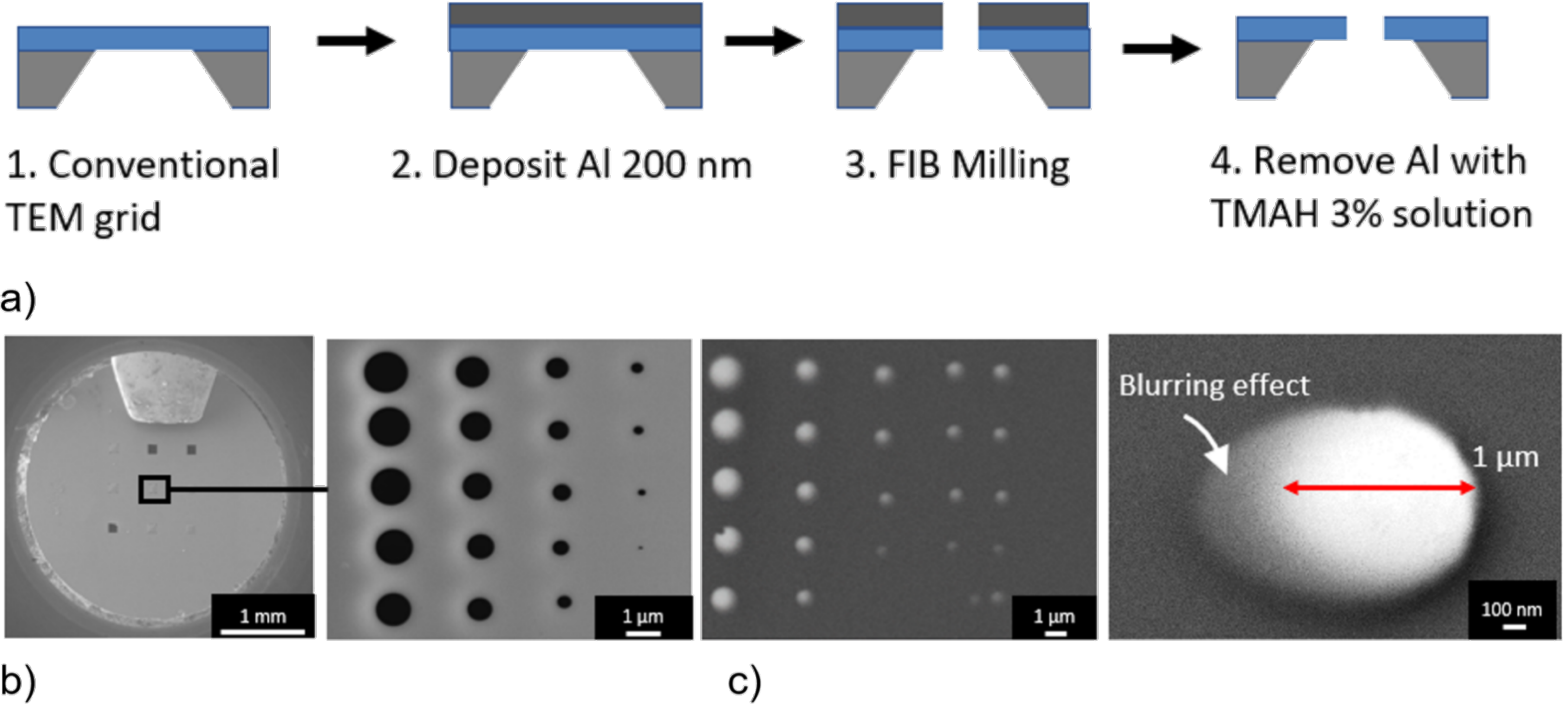
(a) Hard mask preparation. (b) SEM image of the hard mask. (c) SEM images of deposited Py and a 1 µm Py disk.

The mask fabrication process (Figure 9a) is the following: A 200 nm thick sacrificial layer of aluminium was deposited by means of evaporation on a conventional TEM grid with a free-standing SiN membrane. The aluminium layer serves as a support for the free-standing membrane and as a conductive layer for better imaging during FIB milling. Then FIB milling was performed to create apertures in the SiN membrane representing the patterns to be transferred to the sample. Last, the aluminium layer was removed by submerging the mask in TMAH 3% solution. In this project, a TEM substrate with nine free-standing SiN membranes was used as a hard mask. We lay an identical substrate flat on the mask in a flip-chip configuration. The mask is aligned so that all nine windows are on top of each other, and the asymmetrically broken windows serve as an orientation aid. Substrate and mask were fixed using Kapton^®^ foil with an adhesive layer. After deposition, the foil can be removed and the mask can be detached. The mask can still be used, but the resulting nanodots will be smaller than those obtained from the previous deposition because the aperture size on the mask is reduced by residual deposited material.

As seen in Figure 9c, the structure has a blurring effect. One of the sources of blurring in stencil lithography is the geometry of the source–stencil–substrate configuration. The deposited structure is larger than the stencil aperture. Their size difference (the blurring size) is proportional to the size of the source and the distance between the substrate and the mask, and inversely proportional to the sample-to-source distance [30]. It is then required to decrease the distance between the mask and the substrate as well as to employ an evaporation process from a distant point-like source. Furthermore, metal deposits on the mask aperture leads to clogging, causing masks to become ineffective after multiple uses.

### Preparation of nanostructures starting from a bulk substrate

**Figure 10:**
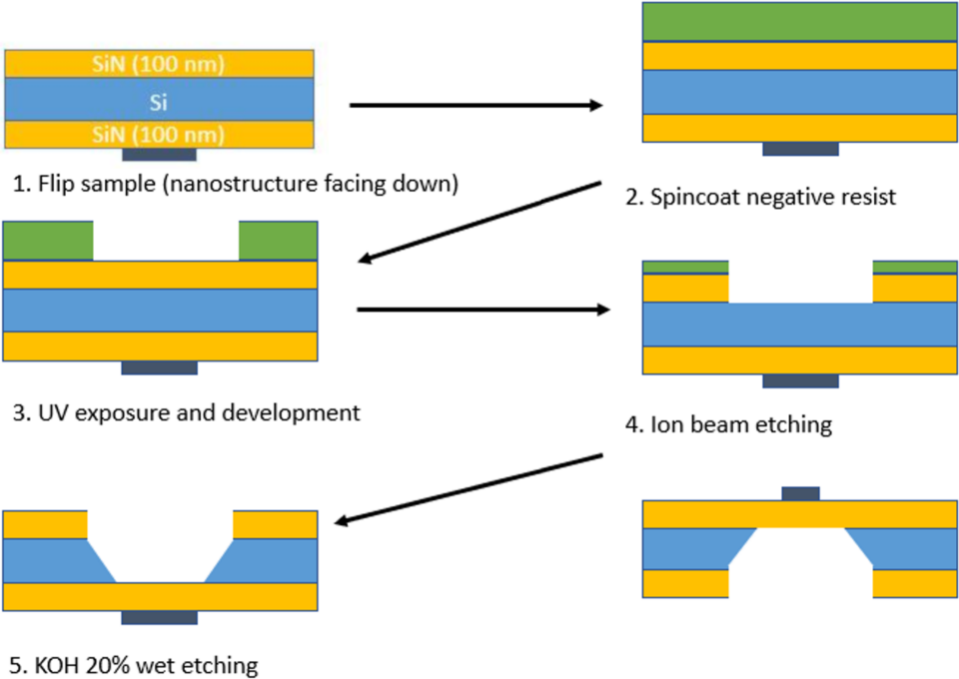
SiN membrane preparation on a nanostructure.

To avoid the delicate procedure of fabricating nanostructures on a free-standing SiN membrane, we initially fabricated the nanostructures on a 200 µm thick Si substrate with 100 nm thick SiN buffer layers on both sides. The buffer layers were deposited with low-pressure CVD to ensure stress-free films. The fabrication process is shown in Figure 10.

First, we made the nanostructure using lift-off or IBE methods on the bulk substrate. Then we protected the nanostructure with a resist and patterned AZ^®^5214E resist on the back side of the substrate using the image reversal technique. A window in the SiN buffer layer was prepared by ion beam etching through the aperture in the mask of AZ^®^5214E resist, and the remaining SiN layer served as a hard mask for the wet etching of the substrate’s back side in 20% KOH solution. The KOH solution was heated to 60 °C to accelerate the process to an etching rate of approximately 9 μm/h in the direction perpendicular to the substrate surface. Using a warmer KOH solution resulted in a faster etch rate but led to much stronger bubbling and roughening of the Si surface with the creation of micropyramidal hillocks [31].

**Figure 11:**
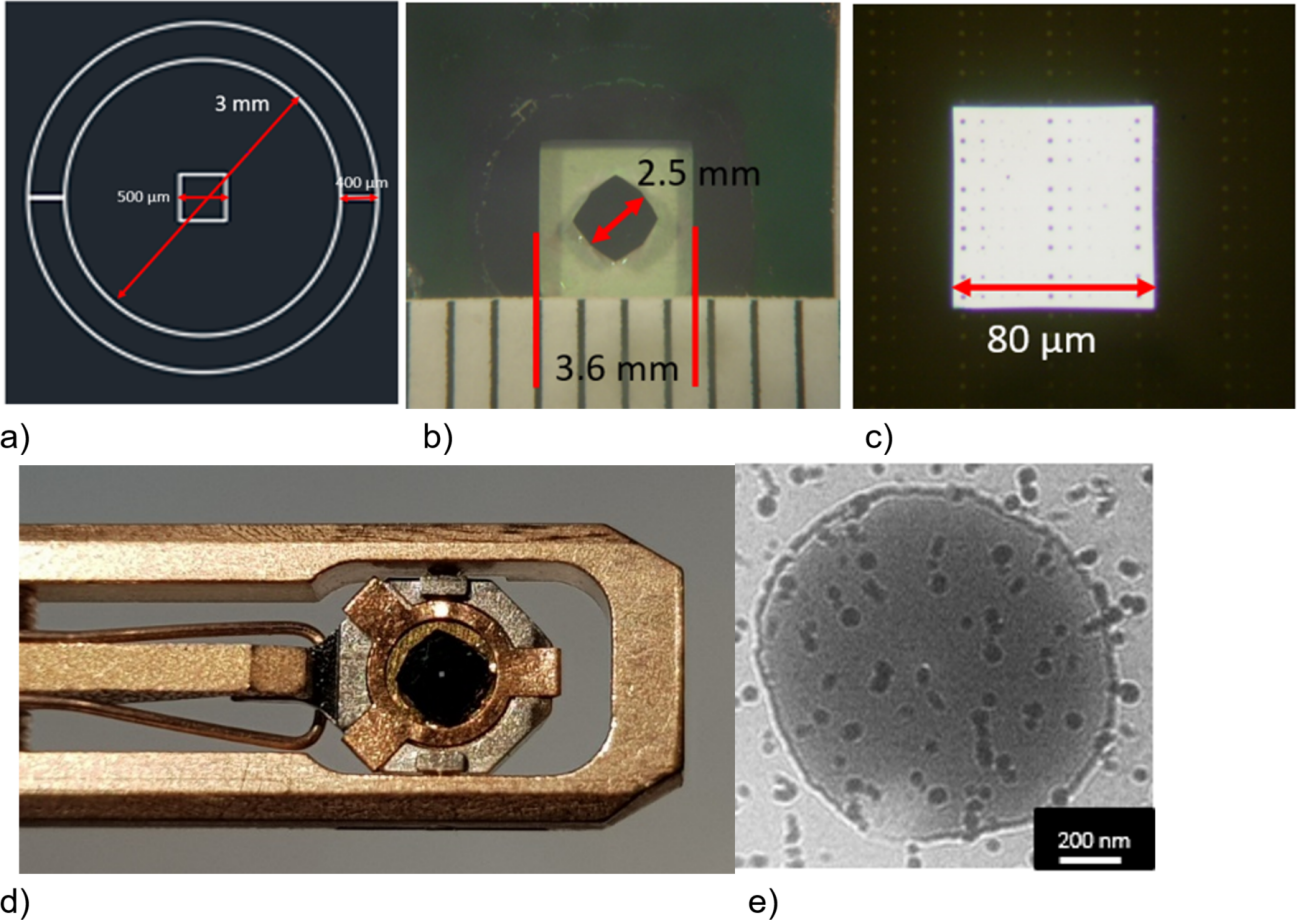
(a) Lithography mask. (b) Etched substrate still attached to the bulk substrate. (c) Free-standing SiN membrane. (d) The TEM substrate glued to a 3 mm ring is mounted on the TEM holder. (e) LTEM image of a 1 µm Py disk on a SiN membrane. The small dots are residuals of the protective resist layer.

The surface alignment of the Si substrates is parallel to the {100} crystallographic plane of Si, and the anisotropic KOH etching results in a 54.7° slope with respect to the etched surface on the sidewalls. Because of the (010) edge orientation of Si substrates, etching occurs faster in diagonal directions of the substrates, resulting in sharp edges on the structure (Figure 11b).

### Magnetic measurements

#### Setup

**Figure 12:**
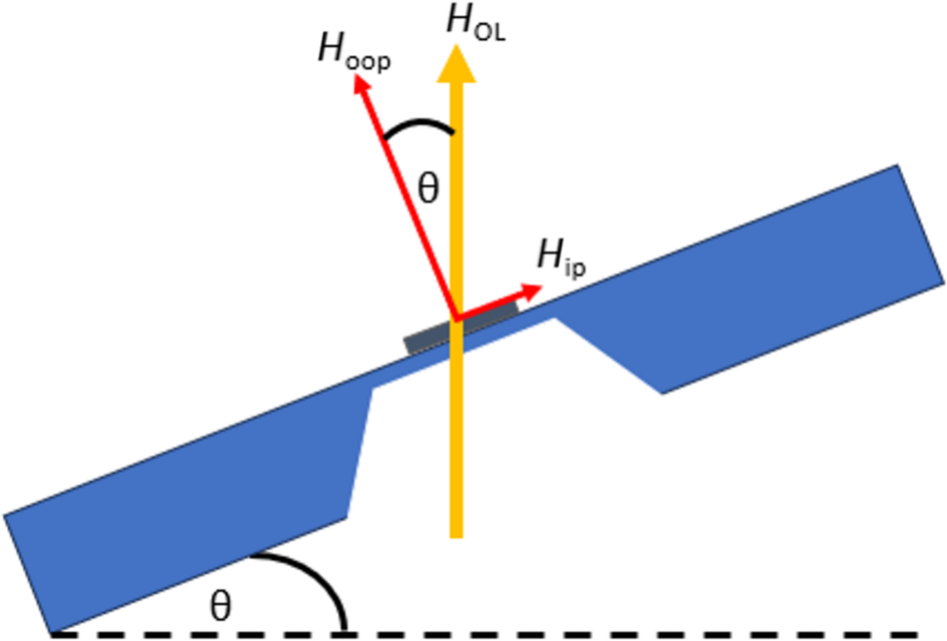
Tilted sample and the external magnetic field component.

All TEM magnetic measurements were performed using an FEI Titan HOLO G2 60-300 microscope with an acceleration voltage of 300 kV with two biprisms. The microscope was used in the Lorentz mode, which gives a spatial resolution up to 2 nm. The sample was mounted on a standard double-tilt holder and inserted into the microscope. The magnetization of the sample was first saturated out-of-plane by switching on the objective lens of the TEM and then allowing it to relax. To switch the magnetic configuration, a small objective-lens field was applied, which resulted in in-plane (*H*_ip_) and out-of-plane (*H*_oop_) fields applied to the sample (Figure 12) depending on the sample tilt. We kept the tilt angle constant and applied different intensities of the objective-lens field. The magnetic states of the sample were characterized under each condition using LTEM and off-axis electron holography.

#### Lorentz TEM

**Figure 13:**
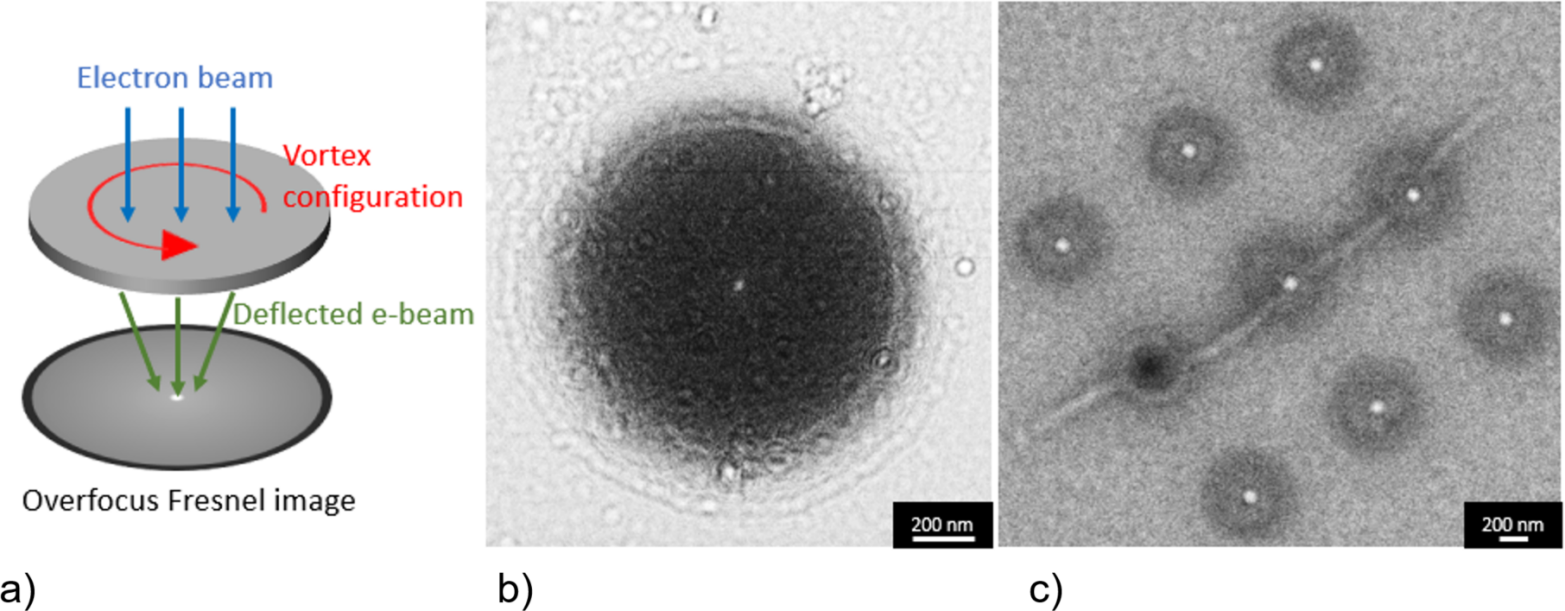
(a) Simplified illustration of the LTEM technique. (b) LTEM image of a 1 µm Py disk. (c) LTEM image of a 400 nm disk array.

The dynamics of the magnetic vortex with external magnetic field applied using the objective lens was first studied using Lorentz TEM. The initial magnetic configuration of the Py nanodisks after relaxation is shown in Figure 13c. In order to ascertain the coercive field of the sample, we applied the external field until the contrast was no longer visible, which means we saturated the magnetic components until all pointed to the same in-plane direction. The motion of the vortex with the field was studied using the following field sequence. First, we saturated the magnetic configuration by applying a large field. Then we decreased the external field until we observed the vortex followed by increasing the field in the opposite direction. In the end, we decreased the field to zero. LTEM images were captured at different magnetic fields along this sequence and are presented in Supporting Information File 1.

As the external magnetic field is applied, the vortex core (bright or dark spot) moves perpendicular to the applied field toward the edge of the disk such that the magnetization component aligned with the field grows bigger. The vortex core displacement near zero external field is in linear proportion to the applied magnetic field, which is consistent with prior results [32]. At the saturation field, the vortex core disappears because the magnetization is now in a single-domain state.

When the external magnetic field decreases, the formation of the vortex does not occur immediately, but after the field reaches about 30 mT (nucleation field), the bright spot appears near the edge of the disks. The same observation was made as we applied the opposite field direction until saturation and started decreasing it. The saturation field is larger than the nucleation field, which agrees with other works [7–8,33].

#### Off-axis electron holography

**Figure 14:**
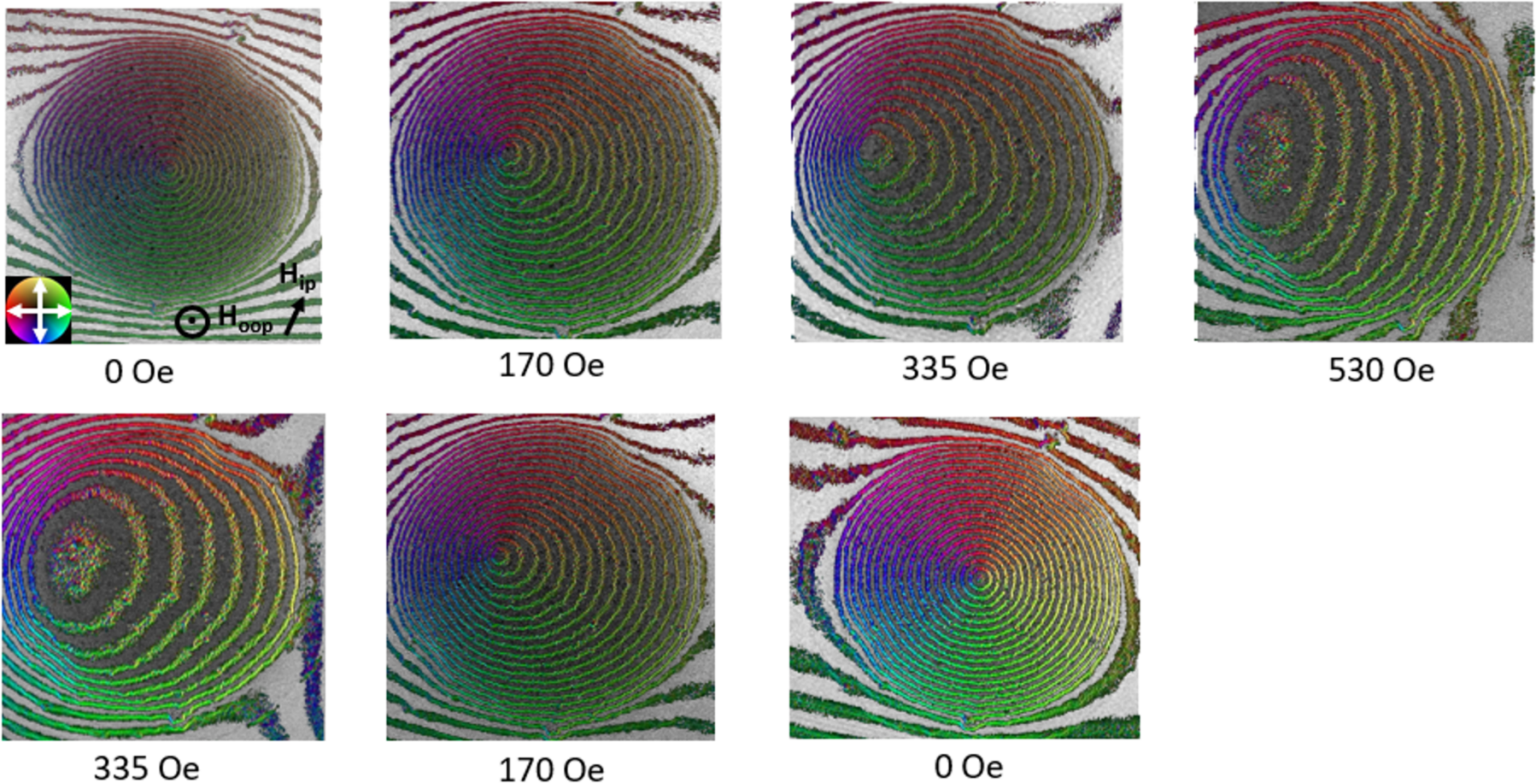
Magnetic induction map of a nanodisk under various magnetic fields obtained by off-axis electron holography. The fringes outside of the disk are probably caused by the charging of the SiN membrane.

The limitations of LTEM are that (1) the magnetic contrast is visible only under defocused conditions, which significantly limits the spatial resolution, and that (2) magnetization information is not quantitative unless multiple LTEM images are taken at different focuses for further reconstruction using the transport of intensity equation. Using off-axis electron holography, we were able to directly measure the phase shift induced by the in-plane magnetization in focus and quantitatively. Using the same parameters as in the LTEM experiment, we kept the tilt angle of the sample constant and started with increasing the external field from 0 to 530 Oe (Figure 14 upper row), which is bigger than the saturation field we observed during the LTEM experiments. Then, we decreased the magnetic field back to 0 Oe (Figure 14 bottom row). We observed the movement of the vortex core with an applied objective-lens field. The vortex state started with counterclockwise helicity and moved perpendicular to the applied field. The magnetization before and after saturation was not similar under an external field of 335 Oe since nucleation started after the external field decreased to around 300 Oe. The results are consistent with the observations from LTEM.

## Conclusion

Electron beam lithography in combination with lift-off and IBE methods for the preparation of Py nanodisks has been optimized. The results have been compared using SEM and TEM. In addition, the application of stencil lithography for the preparation of Py nanodisks has been demonstrated, and the results were compared with the results obtained with electron beam lithography.

Among these methods, dry etching stands out for its sharp and well-defined edges, while the hard mask method offers the least precise results. In a broader comparison, IBE outperforms the lift-off technique, delivering cleaner and more reproducible nanostructures with better spatial resolution. Lift-off fabrication allows for the creation of nanodisks with dimensions ranging from 400 nm to 1.6 µm, but as the size decreases, structural precision diminishes. In contrast, dry etching can yield nanodisks as small as 200 nm with high precision. However, it is important to consider that the IBE process may result in thinner SiN membranes, potentially compromising their mechanical stability. Furthermore, IBE, which is a physical etching process, redeposits a non-volatile metal layer on the resist, creating fences on the edge of the structure and contaminating the silicon nitride membrane. A possible solution is replacing IBE with reactive ion etching (RIE). Using RIE, there would be less redeposition since the reaction between gas and etched metal will form a gaseous compound or volatile particles, which can be pumped out. RIE of nickel–iron alloys has been carried out using inductively coupled plasma RIE with argon and chlorine [34] or with NH_3_ and CO [35]. Stencil lithography requires further optimization although possible applications are attractive because of the flexibility of the patterns, reusable masks, and resistless fabrication. This would be useful because TEM grids are too small for standard lithography. After all, spin-coated resist on TEM grids has inhomogeneous thickness because of edge beads, which become too large relative to the diameter of the TEM grid. Alignment of the stencil mask in a flip-chip configuration with the sample under clean room conditions prior to metal deposition plays an important role as a single dust particle can increase the gap between the mask and the sample up to about 100-fold, thereby increasing the shadowing effect greatly.

KOH etching opens further applications for TEM sample preparation for more complicated high-resolution nanostructures. We have developed a straightforward method to prepare a SiN membrane with nanostructures on one side. This method allows for the use of an ultrasonic bath, higher deposition temperatures, and a homogeneous resist layer, all of which are difficult to obtain with free-standing SiN membranes, resulting in more reproducible results. In principle, some characterizations that do not require an electron-beam-transparent membrane can be done while the nanostructures are on a bulk substrate. Once all measurements have been completed, KOH etching can be conducted, and as a result, the sample is placed on a free-standing SiN membrane and can be studied under TEM. This is useful for future high-frequency correlative characterization of multilayer spintronic devices. Another possible further development is to use a different membrane, for example SiC (lattice constant *a* = 0.435 nm), since it can grow as a single-crystalline layer and ensure epitaxial sample growth on top of it, for example, the growth of NbN (*a* = 0.439 nm) with a lattice mismatch of 1%. Epitaxial growth of Py films on single-crystal SiC membranes is also feasible. Py epitaxial films were obtained on single-crystal MgO substrates [36] that have a lattice constant of 0.42 nm. It was demonstrated that the epitaxial SiC layer can serve as an excellent mask material for KOH etching of Si [37]. However, etching to a crystalline membrane might be different from etching to a free-standing amorphous SiN membrane. The structural integrity of a free-standing crystalline membrane during KOH etching is still to be investigated. An alternative to KOH etching would be FIB milling [38] or RIE [39].

## Supporting Information

File 1 Video of Lorentz TEM measurements.
